# Protection in Macaques Immunized with HIV-1 Candidate Vaccines Can Be Predicted Using the Kinetics of Their Neutralizing Antibodies

**DOI:** 10.1371/journal.pone.0028974

**Published:** 2011-12-28

**Authors:** David Davis, Wim Koornstra, Daniella Mortier, Zahra Fagrouch, Ernst J. Verschoor, Jonathan L. Heeney, Willy M. J. M. Bogers

**Affiliations:** 1 Department of Virology, Biomedical Primate Research Centre, Rijswijk, The Netherlands; 2 Laboratory of Viral Zoonotics, University of Cambridge, Cambridge, United Kingdom; New York University, United States of America

## Abstract

**Background:**

A vaccine is needed to control the spread of human immunodeficiency virus type 1 (HIV-1). An *in vitro* assay that can predict the protection induced by a vaccine would facilitate the development of such a vaccine. A potential candidate would be an assay to quantify neutralization of HIV-1.

**Methods and Findings:**

We have used sera from rhesus macaques that have been immunized with HIV candidate vaccines and subsequently challenged with simian human immunodeficiency virus (SHIV). We compared neutralization assays with different formats. In experiments with the standardized and validated TZMbl assay, neutralizing antibody titers against homologous SHIV_SF162P4_ pseudovirus gave a variable correlation with reductions in plasma viremia levels. The target cells used in the assays are not just passive indicators of virus infection but are actively involved in the neutralization process. When replicating virus was used with GHOST cell assays, events during the absorption phase, as well as the incubation phase, determine the level of neutralization. Sera that are associated with protection have properties that are closest to the traditional concept of neutralization: the concentration of antibody present during the absorption phase has no effect on the inactivation rate. In GHOST assays, events during the absorption phase may inactivate a fixed number, rather than a proportion, of virus so that while complete neutralization can be obtained, it can only be found at low doses particularly with isolates that are relatively resistant to neutralization.

**Conclusions:**

Two scenarios have the potential to predict protection by neutralizing antibodies at concentrations that can be induced by vaccination: antibodies that have properties close to the traditional concept of neutralization may protect against a range of challenge doses of neutralization sensitive HIV isolates; a window of opportunity also exists for protection against isolates that are more resistant to neutralization but only at low challenge doses.

## Introduction

The infection of rhesus macaques by simian human immunodeficiency virus (SHIV) can be used as a model to study the effects of active and passive immunization [1], [2], [3]. SHIV are chimeric virus which have been engineered with the inner, structural components of simian immunodeficiency virus (SIV) as well as the enzymes required for replication in macaques. In the present study, we have used SHIV_SF162_ where the envelope of HIV-1_SF162_ has been substituted for that of SIV_mac239_ [4]. This chimeric virus has been passaged four times through rhesus macaques [5]. Passive transfer studies indicate that full protection can be obtained with a human monoclonal antibody, IgG1 b12 and challenge with SHIV_SF162P4_ by the intravaginal route [6]. However, complete protection required antibody concentrations which could not reasonably be expected to be induced by available vaccine candidates and current immunization strategies. Similarly, reductions in peak viral load in HIV-1_SF162_ envelope-immunized macaques primed with alphavirus replicon particles and boosted with recombinant glycoprotein correlated with serum neutralizing antibody titers against HIV-1_SF162_ pseudovirus in the TZMbl assay [7].

In previous studies with sera from immunized macaques which were fully protected against SHIV challenge [8], we could not show any neutralization in standard assays against HIV which had been prepared in human peripheral blood mononuclear cells (HIV prepared in PBMCs = primary virus) [9]. Neutralization could only be demonstrated if the incubation phase was extended. However, assays with PBMCs as targets are not sufficiently precise to quantify neutralization kinetics [10]. Assays with GHOST cells offer greater precision [11]. GHOST cells are human osteosarcoma cells which have been engineered to express green fluorescent protein following infection with HIV or SHIV isolates. The cells have also been engineered to display CD4 which is the receptor for HIV and the various chemokine receptors which act as co-receptors. The fluorescent cells can be quantified using a fluorescence activated cell scanner and represent a measure of the number of infectious virus.

The aim of the present study was to quantify various parameters of the neutralization reaction using sera from rhesus macaques which had been immunized with HIV-1 envelope vaccine candidates (immunogens and schedules are summarized in tables 1 and S1). A further aim was to determine if the parameters had any association with protection [12], [13], [14], [15] when the macaques were subsequently challenged with SHIV_SF162P4_. Assay formats with the potential to predict protection are described.

**Table 1:** Summary of sources of sera from immunogenicity trials in rhesus macaques.

Trial	Group	Immunogens	Immunization (weeks)	Challenge
1	1.1	Recombinant SF162 ΔV2 gp140	0, 6, 16, 36, 47	Intravenous: 50 TCID_50_
	1.2	Recombinant SF162 ΔV2 gp140	0, 6	
		SF162 V3 linear peptide	16, 37, 47	
		19b and IgG1 b12 mimotope	16, 37, 47	
	1.3	Recombinant TV1 ΔV2 gp140	0, 6, 16, 36, 47	
	1.4	Recombinant TV1 ΔV2 gp140	0, 6	
		TV1 V3 peptide;	16, 37, 47	
		19b and IgG1 b12 mimotope	16, 37, 47	
	1.5	Controls		
2	2.1	Recombinant 461, SF162 and TV1 gp140	0, 6, 16	Intrarectal: 1,800 TCID_50_
	2.2	Recombinant 461, SF162 and TV1 gp140	0, 6	
		SF162 V3 cyclised peptide	6, 16	
		SF162 V2 linear peptide; IgG1 b12 mimotope	6, 16	
	2.3	Recombinant 461, SF162 and TV1 gp140	0, 6	
		TV1 V3 cyclised peptide	6, 16	
		MPER peptide; IgG1 b12 mimotope	6, 16	
	2.4	Controls		
3	3.1	Recombinant 461, SF162 and TV1 gp140	0, 6, 16	Intrarectal: 1,800 TCID_50_
	3.2	SF162 V3 cyclised peptide	0, 6	
		SF162 V2 linear peptide; IgG1 b12 mimotope	0, 6	
		Recombinant 461, SF162 and TV1 gp140	6, 16	
	3.3	TV1 V3 cyclised peptide	0, 6	
		MPER peptide; IgG1 b12 mimotope	0, 6	
		Recombinant 461, SF162 and TV1 gp140	6, 16	
	3.4	Controls		
4	4.1	Adenovirus Ad5hr-89.6PΔCFI gp140	0, 12	Intrarectal: 1,800 TCID_50_
		Recombinant SF162 gp140	24, 36	
	4.2	Adenovirus Ad5hr-89.6PΔCFI gp140	0, 12	
		VEE replicons encoding SF162 gp140	24, 36	
	4.3	Controls		
5	5.1	VEE encoding SF162 ΔV2 gp140	0, 4, 12	Intrarectal: 120 MID_50_
		Recombinant SF162 gp140	24, 36	
	5.2	VEE encoding MJ4 gp140	0, 4, 12	
		Recombinant MJ4 gp140	24, 36	
	5.3	VEE encoding SF162 ΔV2 and MJ4 gp140	0, 4, 12	
		Recombinant SF162 and MJ4 gp140	24, 36	
	5.4	Empty VEE replicons	0, 4, 12	
		Recombinant SF162 and MJ4 gp140	24, 36	
	5.5	Controls		

## Results

### Neutralizing antibody titers show variable correlation with protection

**Figure 1:**
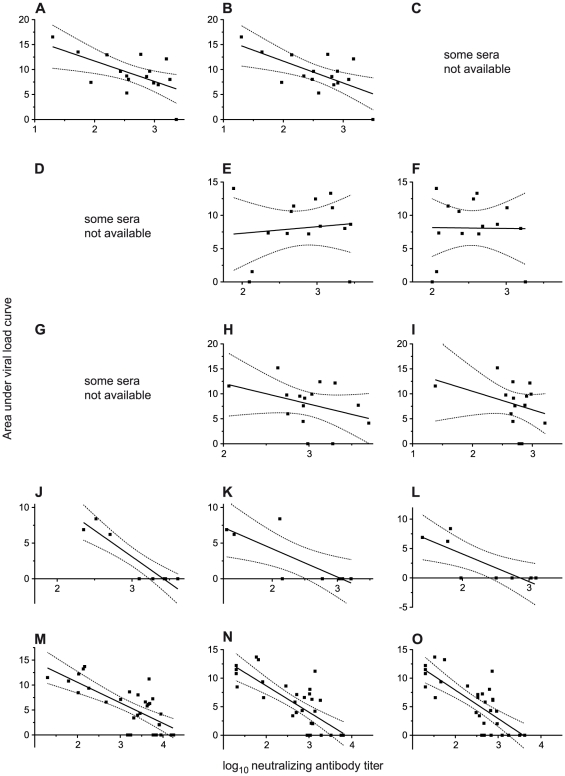
Regression analysis of neutralizing antibody titer with infection after SHIV_SF162P4_ challenge of immunized rhesus macaques. Macaques are challenged 8 weeks after the final immunization. Neutralizing antibody titer is the dilution of serum which gives a 50% reduction in luciferase production in 1/48/2 TZM-bl cells with SHIV_SF162P4_ pseudovirus. Infection is quantified as the area under the plot of viral load against time following challenge for individual macaques. Viral load was measured as log_10_ RNA equivalents per ml of plasma and time in weeks. Regression lines are presented in the form: y = mx + c where y is the area under the viral load curve, m is the gradient, x is the neutralizing antibody titer and c is the intercept. **A**, immunization trial 1; 6 weeks before challenge. Spearman r = −0.5324, p = 0.0338. m = −4.090±1.417, c = 19.90±3.776, r^2^ = 0.3729, p = 0.0120. **B**, immunization trial 1; 2 weeks before challenge. Spearman r = −0.4912 p = 0.0534. m = −4.380±1.370, c = 20.46±3.588, r^2^ = 0.4221, p = 0.0065. **C**, immunization trial 1; sera from some macaques not available at challenge. **D**, immunization trial 2; sera from some macaques not available at 6 weeks before challenge. **E**, immunization trial 2; 2 weeks before challenge. Spearman r = 0.07864, p = 0.7806. m = 0.9875±2.445, c = 5.333±6.947, r^2^ = 0.01239, p = 0.6929. **F**, immunization trial 2 at time of challenge. Spearman r = −0.03932, p = 0.8893. m = −0.1269±3.035, c = 8.418±7.803, r^2^ = 0.0001344, p = 0.9673. **G**, immunization trial 3; sera from some macaques not available at 6 weeks before challenge. **H**, immunization trial 3; 2 weeks before challenge. Spearman r = −0.3056, p = 0.2680. m = −4.134±2,888, c = 20.36±8.716, r^2^ = 0.1362, p = 0.1759. **I**, immunization trial 3 at time of challenge. Spearman r = −0.2987, p = 0.2794. m = −3.705±2.806, c = 17.93±7.616, r^2^ = 0.1182, p = 0.2096. **J**, immunization trial 4; 6 weeks before challenge. Spearman r = −0.8456, p = 0.0107. m = −7.395±1.217, c = 25.29±3.758, r^2^ = 0.8602, p = 0.0009. **K**, immunization trial 4; 2 weeks before challenge. Spearman r = −0.7910, p = 0.0279. m = −3.967±1.236, c = 12.15±3.074, r^2^ = 0.6319, p = 0.0184. **L**, immunization trial 4 at time of challenge. Spearman r = −0.7910, p = 0.0279. m = −4.710±1.449, c = 13.50±3.437, r^2^ = 0.6377, p = 0.0175. **M**, immunization trial 5; 6 weeks before challenge. Spearman r = −0.6537, p<0.0001. m = −4.105±0.7381, c = 18.77±2.437±2.488, r^2^ = 0.5161, p<0.0001. **N**, immunization trial 5; 2 weeks before challenge. Spearman r = −0.6925, p<0.0001. m = −4.629±0.7528, c = 17.94±2.079, r^2^ = 0.5660, p<0.0001. **O**, immunization trial 5 at time of challenge. Spearman r = −0.6941, p<0.0001. m = −4.981±0.8182, c = 17.87±2.089, r^2^ = 0.5610, p<0.0001.

Some macaques were completely protected while others which had equal or greater *in vitro* neutralizing antibody titers became infected (Figure 1). Neutralization antibody titers in the 1/48/2 TZMbl *in vitro* assay with sera from the different challenge studies showed a variable capacity for predicting protection. This variability was highest between immunization strategies: regression coefficients varied between −0.03932 in trial 2 and −0.8456 in trial four. In contrast, coefficients were relatively consistent at different times prior to challenge within each trial. Subsequent studies used sera, with neutralization titers of approximately 1 in 1,000 or greater, taken from the macaques two weeks before challenge. This titer of neutralizing antibodies may be expected to influence *in vivo* protection. Various scenarios can be proposed to explain the variability in predictive capacity of the TZMbl assay. Neutralizing antibodies may not themselves be protective but their levels reflect some other, protective immune response. It is also possible that the antibodies which are being detected at the highest dilution with *in vitro* assays are not the ones which are protective *in vivo*. We may need to quantify neutralization at lower dilutions.

### Rates of neutralization can be quantified using GHOST cells

**Figure 2:**
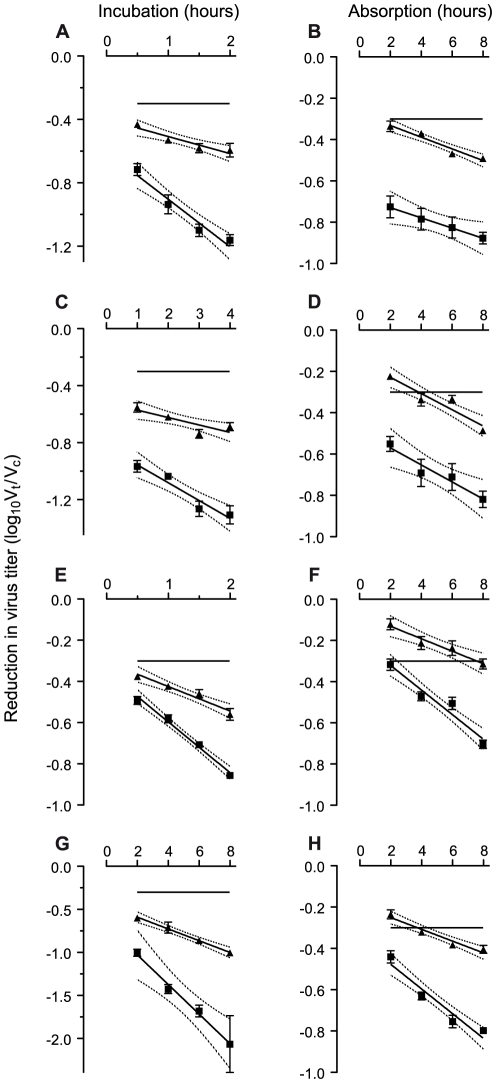
Reductions in infectious titer following exposure of HIV-1_SF162_ to sera from immunized rhesus macaque. Sera are taken two weeks before challenge. Reductions in infectious virus are calculated as ratio of the titer (V_t_) at time t for the virus exposed to serum from an immunized macaque divided by the titer (V_c_) at the same time for a control serum. The ratio is transformed to log_10_ (V_t_/V_c_). Incubation and absorption phases are measured in hours. Data are displayed as means with standard errors. Plots are regression lines with 95% confidence band. Solid squares: 1 in 20 serum dilution; solid triangles: 1 in 50 serum dilution. Expected ratio of neutralization rates is the ratio of the serum concentrations within an individual assay: 2.5. **A**, Incubation plots of serum from protected macaque in treatment group 3.2 (Ratio = 2.79; p = 0.0004189); **B**, absorption plots from same macaque (Ratio = 0.88; p = 0.7321); **C**, Incubation plots from protected macaque in treatment group 4.1 (Ratio = 2.40; p = 0.01276); **D**, Absorption plots from same macaque (Ratio = 1.05; p = 0.8763). **E**, Incubation plots from infected macaque in treatment group 2.1 (Ratio = 2.07; p<0.0001). **F.** Absorption plots from same macaque (Ratio = 1.97; p = 0.00305). **G**, Incubation plots from infected macaque in treatment group 2.3 (Ratio = 2.52; p = 0.00842). **H.** Absorption plots from same macaque (Ratio = 2.10; p = 0.0002994).

GHOST cells fluoresce when infected with HIV-1 and can be used to quantify individual infectious events. Neutralization of HIV-1_SF162_ by sera from immunized macaques in a/24/2 (for explanation see Materials and Methods section) GHOST assays was exponential (equal proportions of virus are neutralized per unit of time following exposure to antibody.) Neutralization rates could be distinguished at different serum dilutions (Figure 2A, C, E, G). However, it is apparent that if the plots are extrapolated back to zero time ( = the intercept, where the line crosses the vertical or y-axis), they do not pass through the origin (point 0, 0 where the vertical and horizontal axes cross): there is significant neutralization (>50%) without any incubation. As the virus is slow to bind to the target cells, this neutralization may be the result of antibody binding to free virions in the supernatant above the target cells. Alternatively, the presence of cells may be obligatory and events following the exposure of virus or virus-antibody complexes to targets may determine the eventual extent of neutralization.

### Level of neutralization increases as absorption phase is extended

Exponential neutralization was also seen during the absorption phase of a 1/b/2 GHOST assay (Figure 2B, D, F, H). An absorption phase is required in any assay but by extrapolating the plots back, the point at which they cross the vertical axis can give a measure of the neutralization at zero time of absorption which also corresponds with the end of the incubation phase.

### Neutralization is not exponential during the incubation phase

**Figure 3:**
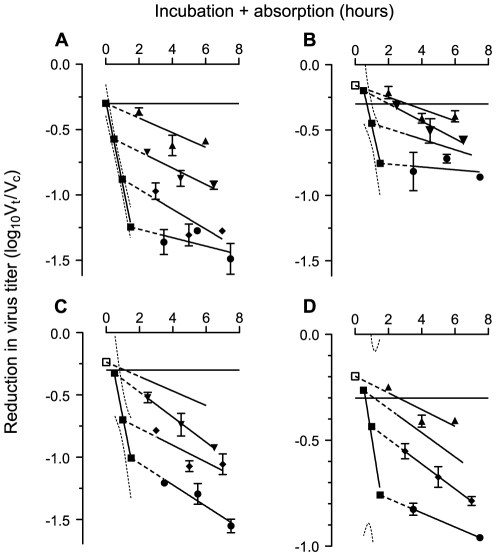
Incubation plus absorption plots of HIV-1_SF162_ neutralization with sera from protected macaques. Sera are taken two weeks before challenge. Reductions in infectious virus are calculated as ratio of the titer (V_t_) at time t for the virus exposed to serum from an immunized macaque divided by the titer (V_c_) at the same time for a control serum. The ratio is transformed to log_10_ (V_t_/V_c_). Incubation and absorption phases are measured in hours. Data are displayed as means with standard errors. Solid horizontal line represents 50% neutralization. Triangles, diamonds and discs: regression lines for absorption plots following incubation for different intervals. Some symbols are excluded to improve clarity. Intercepts determined (straight dotted lines) giving reduction in virus titer when absorption is zero (≡ end of incubation phase) and plotted as solid squares. Regression line with 95% confidence band (curved dotted lines). Open squares are data which have been excluded from calculation of regression line. **A**. Macaque in treatment group 2.1: 1 in 40 dilution of serum: Reduction during incubation phase of log_10_ 0.6292±0.02930 infectious doses per hour starting at log_10_ −0.3948 to −0.1589 (95% confidence interval) infectious doses. r^2^ = 0.9957; p = 0.0022 **B.** Serum from same macaque at 1 in 100 dilution: reduction rate log_10_ 0.5569±0.03193 infectious doses per hour starting at log_10_ −0.3479 to 0.5284 infectious doses. r^2^ = 0.9967; p = 0.0365 **C** and **D** Sera at 1 in 20 dilution from macaques in treatment group 3.2. Reduction rate for C is log_10_ 0.6812±0.04007 infectious doses per hour starting at log_10_ −0.5457 to 0.5541 infectious doses; r^2^ = 0.9966; p = 0.0374 Reduction rate for D is log_10_ 0.4948±0.08753 infectious doses per hour starting at log_10_ −1.192 to 1.210 infectious doses; r^2^ = 0.9697; p = 0.115.

Plots where both incubation and absorption phases were varied (Figure 3) indicate that there was a delay before the inactivation of free virions enters its exponential phase (Figure 3 B, C, D). Although neutralization with the serum of one protected rhesus macaque appeared to be exponential (Figure 3A), when the serum was diluted (Figure 3B), there was a delay. The intercepts of the exponential phases of all three sera are close to the origin.

### Neutralization rates do not correlate with protection

**Figure 4:**
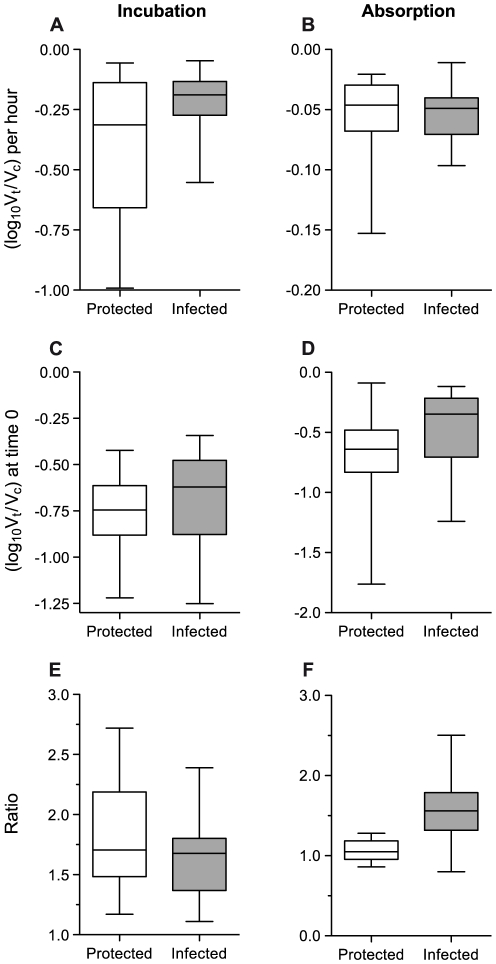
Comparison of serum neutralization functional properties between protected and infected immunized rhesus macaques. Data are summarized in box and whisker plots of the gradients of regression lines (neutralization rates: log_10_ (V_t_/V_c_) per hour) and the intercepts (log_10_ (V_t_/V_c_) at time 0) with a 1 in 20 dilution of serum. Also, the ratios of neutralization rates with different serum dilutions (expected ratio = 2.5) are displayed. The medians of protected (n = 13; open boxes) and infected (n = 22; striated boxes) rhesus macaques are compared by the Mann-Whitney non-parametric two sample test. Neutralization rates during **A**, incubation phase (p = 0.0788) and **B**, absorption phase (p = 0.7457) of neutralization assay; intercepts during **C**, incubation (p = 0.1888) and **D**, absorption (p = 0.1125) phase of assay; **E**, incubation ratios (p = 0.3748) and **F**, absorption ratios (p = 0.0004).

The neutralization parameters were determined using GHOST cell assays for sera from protected (n = 13) and infected (n = 22) macaques. It seemed reasonable that a neutralization titer of approximately 1 in 1,000 or greater in the TZM-bl assay had the potential to influence *in vivo* protection. There was no statistically significant difference between the rates of neutralization (Figure 4 A, B) of sera from protected and infected macaques during either the incubation (p = 0.0788) or the absorption (p = 0.7457) phases. Similarly, the intercepts also showed no statistically significant differences (p = 0.1888 for the incubation phase and p = 0.1125 for the absorption phase (Figure 4 C, D).

### Ratios of neutralization rates at different serum dilutions are lower than expected

**Figure 5:**
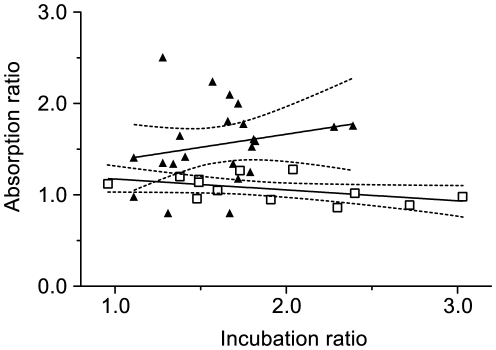
Regression lines of incubation ratios against absorption ratios. Regression lines are plotted for the protected (n = 13; open squares) and infected (n = 22; triangles) immunized rhesus macaques. Strictly speaking, as neither the incubation ratio nor the absorption ratio can be considered the independent variable and neither has a fixed value, a regression analysis is not legitimate. None the less, for the reader's convenience, we present linear regression lines in the form of y = mx + c: Protected macaques: m = −0.119±0.061; c = 1.293±0.121; r^2^ = 0.2557; p = 0.0779. Infected macaques: m = 0.287±0.290; c = 1.090±0.477; r^2^ = 0.04685; p = 0.3333. The gradients of the lines are not statistically significantly different (p = 0.1785). in contrast, the intercepts are highly significantly different (p = 0.0007586).

The ratio of the serum dilutions (e.g. 1 in 20 and 1 in 50 in Figure 2) in the assays was 2.5. However, the ratios of the rates of neutralization at any two serum dilutions were reduced below their expected values for both the incubation (Figure 4E) and absorption (Figure 4F) phases. There was no statistical significance between the protected and infected macaques for the neutralization rate ratios during the incubation phase. However, in marked contrast, there were statistically significant differences between the neutralization rates of the serum dilutions during the absorption phase for individual macaques (Figure 2 F, H). The difference between the absorption phase ratios for the protected and infected macaques was statistically significant (Figure 4F; Figure 5). During the absorption phase the ratio was one for the protected macaques (Figure 2B, D; Figure 4F). An alternative presentation of the data (Figure 5) also indicates that the dilution of serum from a protected macaque has no influence on the rate of neutralization during the absorption phase independently of its activity during the incubation phase.

### Neutralization of low doses of heterologous virus

Recognition that events during the absorption phase, in addition to those due to the incubation phase of HIV-1 neutralization assays, produce significant effects leads to further conjectures. Firstly, only a fixed number of viruses may be completely inactivated before the target cells remove them or their complexes from the mixture. This contrasts with the proportion of virus which is expected to be inactivated in the reversible reaction between antibody and free virions. Second, assays with formats where the influence of events during the absorption phase is magnified, relative to those during the incubation phase, may reveal neutralization activity against a wider range of isolates, particularly those which are relatively resistant to neutralization.

**Figure 6:**
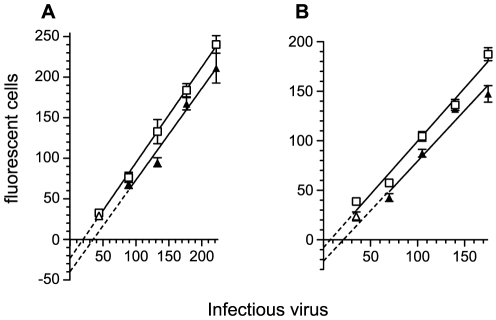
Neutralization of HIV-1_89.6_ by sera from macaques immunized with HIV-1_SF162_ immunogens. Low doses of the relatively neutralization resistant, subtype B HIV-1_89.6_ isolate were incubated at 37°C for four hours with a 1 in 20 dilution of either a control serum (open squares) or serum from a macaque (solid triangles) immunized with HIV-1_SF162_ recombinant immunogens. (Open triangles represent data sets close to background levels which have been excluded from the analysis of regression lines). The mixture was then added to GHOST cells and allowed to absorb for 24 hours. The cells were washed and cultured for a further 24 hours. Results are plotted with regression lines; parameters are given as means with standard errors **A**. Controls: m = 1.179±0.063 fluorescent cells/infectious dose of virus; ; c = −23.33±9.142 fluorescent cells; 95% confidence interval for x-intercept = 3.816 to 32.93 infectious virus doses; immunized macaque from group 2.1: m = 1.129±0.112 fluorescent cells/infectious dose of virus; c = −39.76±18.21 fluorescent cells; 95% confidence interval for x-intercept = 0.7662 to 58.31 infectious virus doses:. The difference between the x-intercepts = 15.6 infectious virus doses. The difference between the gradients was not statistically significant (p = 0.6896) so that the data were pooled and a common gradient calculated: 1.16188. The intercepts on the vertical axis are significantly different (p = 0.001359). **B**. Controls: m = 1.075±0.053 fluorescent cells/infectious dose of virus; ; c = −7.975±6.153 fluorescent cells; 95% confidence interval for x-intercept = −5.086 to 17.81 infectious virus doses; immunized macaque from group 5.1: m = 1.025±0.083 fluorescent cells/infectious dose of virus; c = −23.13±10.64 fluorescent cells; 95% confidence interval for x-intercept = 0.3555 to 38.59 infectious virus doses:. The difference between the x-intercepts = 15.1 infectious virus doses. The difference between the gradients was not statistically significant: p = 0.6064 so that the data were pooled and a common gradient calculated: 1.05833. The intercepts on the vertical axis are significantly different (p<0.0001).

Eight sera with high neutralizing activity against SHIV_SF162P4_ were selected and used in 4/24/2 GHOST assays against a range of low doses of HIV-1_89.6_ (Figure 6). None of the sera significantly changes the gradient of its plot while results from seven are sufficient to produce statistically significant intercepts (Table 2). Although the intercepts are the parameter which achieves formal statistical significance, this may not reflect the real situation. The analysis requires that if the gradients of the plots are not significantly different the data are pooled and a common gradient calculated. This operation does not change the mid points of the plots but can have a considerable influence on the intercepts. Nevertheless, for at least two sera (Figure 6) the difference between the gradients of their plots and controls is less than 5% while the interval between the x-intercepts (points where plots cross the horizontal axis) of their untransformed plots is more than 15 infectious doses. Thus, percentage neutralization increases from low levels with relatively high doses of virus to reach 100% at the lowest doses. It is likely that two neutralization mechanisms are involved. These will be distinguished as virion-associated neutralization producing a percentage reduction in virus titer following events during the incubation phase and cell-associated neutralization producing a fixed reduction due to events during the absorption phase of the assay.

**Table 2:** Neutralizing parameters for selected sera against low doses of the heterologous HIV-1_89.6_ primary isolate.

Group	Gradient†	Intercept¶	% neutralization§	Difference in x-intercepts#
Controls:	1.179±0.063	−23.33±9.142		
2.1	1.285±0.086	−30.33±12.89	−9.00	3.80
2.1	1.129±0.112	−39.76±18.21**	4.07	15.6
Controls	1.075±0.053	−7.975±6.153		
5.1	1.025±0.083	−23.13±10.64***	4.65	15.1
5.4	0.9000±0.080	0.3500±9.340*	16.3	−7.81
Controls	1.080±0.100	−7.903±10.84		
5.3	1.299±0.137	−2.963±14.92**	−20.3	−5.03
5.4	1.108±0.097	2.928±10.56*	−2.59	−9.96
Controls	1.023±0.122	−2.080±12.80		
3.2	0.7449±0.087	2.998±8.791**	27.18	−6.06
3.1	1.302±0.117	−10.04±12.04*	−27.27	5.68

† Number of fluorescent cells/dose of infectious virus.

¶ Number of fluorescent cells (point where plot crosses vertical axis).

§ Gradient of plot with serum from immunized macaque divided by gradient of plot with control serum expressed as a percentage.

# Dose of infectious virus (interval between points where plots for sera from immunized and control macaques cross horizontal axis).

Significant difference with controls:

***p<0.001;

**p<0.01;

*p<0.05.

## Discussion

The traditional concept of neutralization needs to be modified, particularly for the more resistant HIV-1 isolates, in at least two respects: the target cells are not just passive indicators of virus infectivity but are actively involved in the neutralization reaction; the number, rather than the proportion, of viruses inactivated is the feature with more relevance to protection under the conditions found in natural transmission. Both criteria influence the ability of *in vitro* results to predict *in vivo* protection.

The traditional concept of neutralization is that antibody binds to virus and inactivates it [16]. The results from the present and previous studies [10], [17], [18] indicate that multiple steps are involved in HIV-1 neutralization so that the virus-antibody complex remains infectious. Antibody binds to the free virion since neutralization increases as the incubation phase is extended. However, events which occur after the complexes are exposed to the target cells have a major influence on the number of virus eventually inactivated. The reaction between virus and antibody is generally considered to be reversible [16] so that at equilibrium a proportion of virus will be inactivated. In contrast, the reaction which follows the exposure of the complexes to target cells is limited by the removal of the virus and complexes from the surface of the cells. It seems likely therefore that a fixed number of virus will be inactivated. In the present study, we have tried to separate the virus-antibody complex and the cell-associated reactions. The number of virions inactivated is small. Binding of HIV to GHOST cells is slow relative to binding to human PBMCs [17] and is likely to influence the neutralization reaction. Previous studies by ourselves and others indicate that neutralization shows considerable variation when different target cells are used [19], [20], [21], [22], [23], [24], [25]. The present study indicates that the target cells have a decisive influence on the capacity of the *in vitro* assay to predict *in vivo* protection.

Virus, antibody and target cells should be considered as a single system. The combination which comes closest to the traditional concept of neutralization is associated with *in vivo* protection: antibody binds to a virus which is relatively sensitive to neutralization; events which follow the binding of the complex to a target cell inactivate the virus in a reaction which is independent of the antibody concentration. In the present study, antibodies from an immunized macaque may bind to the free virions but if the reaction following exposure to target cells is influenced by the antibody concentration, the virus can still replicate *in vivo*. Virus replication *in vivo* may be slowed but sterilizing immunity is not seen.

The variation in the capacity of the TZMbl assay [26], [27], [28], [29] to predict *in vivo* protection may be attributed to at least two of its features: the 50% neutralization titer can not distinguish between antibodies which inactivate the virus and those which can only slow down virus replication or some other feature associated with viral pathogenicity [17], [30], for example the time taken for the virus-antibody complex to be removed from the surface of the target cell. Increasing concentrations of antibodies which can slow down virus replication may reduce or delay the plasma viremia *in vivo* but antibodies which completely inactivate the virus may be required for sterilizing immunity. It is also possible that *in vivo* protection is determined by the size of the virus inoculum.

There are two ways of quantifying *in vitro* reductions in virus infectious titer. An aliquot of virus can be mixed with antibody and incubated. Then, the virus which remains infectious can be quantified. If 1,000 TCID_50_, for example, were reduced to 200, then this represents 80% neutralization. In our previous studies with human PBMCs [10], [17], [18] or lymphocyte-dendritic cell co-cultures [9], we have used an alternative assay format. We diluted the virus first and added aliquots of serum to the individual doses, incubated the mixtures and determined which dilutions retained infectivity. In this format we are determining the dilution of virus which contains a single infectious dose. Under the equivalent conditions to the example above the dilution originally containing 5 TCID_50_ would be reduced to 1 TCID_50_. This reduction can be considered as 80% neutralization. However, it can also be a reduction of 4 TCID_50_. The formats of the assays used in the present study can distinguish between these two measures: for example is 20 TCID_50_ reduced to 4 TCID_50_ ( = 80% neutralization) or 16 TCID_50_ ( = inactivation of 4 TCID50)? The present study indicates that both percentage reductions and fixed measures contribute to the neutralization reaction with primary isolates of HIV-1. We speculate that antibody binds to the free virus during the incubation phase influencing the percentage of virus eventually inactivated; events during the absorption phase contribute to cell-associated neutralization of a fixed number of virus. Assay formats can be modified so that one measure can be magnified at the expense of the other. Assays with a format measuring reductions in the number of infectious virus may better predict the outcome of human vaccine trials than percentage neutralization. A further conjecture is that there is an interaction effect: antibody binding to the free virus facilitates cell-associated neutralization. However, the GHOST assay is not sufficiently precise to investigate this aspect with the polyclonal sera induced following vaccination.

Quantifying individual infectious events was crucial to testing the mathematical model proposed by Scott Layne [31] and his colleagues for inhibition of HIV infection by soluble CD4. The model shares some features with the explanations offered for the results in the present article. In particular, both systems are a mixture of competitive and irreversible reactions. The model is described by four ordinary differential equations involving five reaction rate constants: the rate of infection of HIV for a particular cell type; the forward and reverse rates of CD4 binding to envelope glycoprotein gp120; the rate of gp120 shedding from virions; the rate of non-specific inactivation of HIV. For the most part, the model meets its design criteria. However, discrepancies between the expected and observed results indicated that some further refinements were still required. The hypothesis was formulated that when HIV virions were incubated in increasing concentrations of soluble CD4 they reached a critical condition in terms of the number of gp120 molecules available for infection. At the same time, soluble CD4 had a threshold concentration for inactivation. Experiments showed that below the threshold, virus inactivation did correspond to the association constant of the CD4-gp120 reaction so that inactivation rates were proportional to soluble CD4 concentration. However, at higher concentrations this relationship was lost and the virus entered a state where it was unduly sensitive to inactivation. It may be speculated that sera from immunized macaques which were not fully protected from SHIV challenge, where the neutralization rate during the absorption phase was also seen to increase with serum concentration, could not induce an equivalent effect. In contrast, antibodies from the protected macaques may have reduced the availability of envelope glycoporteins below a critical level. A further modification of the model which is also pertinent to the present study was to separate the rate of infection into rates for before ( = incubation phase) and after ( = absorption phase) binding of virus to its target cell. Nonetheless, despite these points of agreement, some caveats also need to be acknowledged. The test of the model was performed with virus which had been prepared in continuous cell lines ( = laboratory isolate). Since the work was published, the relevance of primary isolates has been recognized. Isolates which are grown in PBMCs ( = primary cells) may better represent the virus involved in natural transmission events. Primary and laboratory isolates have different properties. In the present context, the most relevant observation is that sera from volunteers in human vaccine trials were able to neutralize laboratory isolates but not primary isolates in conventional assays [32]. It may be that the ratio of the critical level of available infection sites to the actual number of physical sites is greater for the primary isolates. An additional rider relates to the range of available target cells. A detailed description of the molecular changes which follow virus binding to its CD4 receptor has become available [33], [34] since the mathematical model was first formulated. These studies revealed that changes in conformation of the envelope glycoprotein either form, stabilize or reveal a site which is specific for a chemokine receptor and acts as a co-receptor for HIV infection. It may be that the density of these co-receptors, CCR5 for primary isolates and CXCR4 for laboratory isolates, or even the receptor : co-receptor ratio, on the target cells is the limiting factor within the system [24].

Assays which simulate natural transmission events are more likely to predict the outcome of human trials. The dose of virus transmitted may vary with the route of transmission [35], [36], [37], [38]. In particular, higher doses of virus may be transmitted where blood is transferred as with intravenous drug users. Conversely, low doses can be expected by the heterosexual route since natural defense barriers reduce the effective dose of the inoculum. Similarly, the relative resistance to neutralization of the transmitted virus may also influence the results of *in vitro* assays and *in vivo* protection [39], [40], [41], [42], [43]: transmission by intravenous or intrarectal routes avoids natural barriers and may be less selective, allowing more neutralization resistant isolates to infect. In the present study, we have shown that antibodies raised to one subtype B HIV-1 isolate (HIV-1_SF162_), by a vaccine which would be acceptable for human use, can neutralize a relatively resistant heterologous subtype B isolate (HIV-1_89.6_) but only at low doses.

So far we have been able to demonstrate a qualitative effect of neutralization with low doses of virus. For future studies, if we wish to more accurately quantify the number of infectious virus inactivated we need to refine the assays further. This would best be done using monoclonal antibodies which are protective *in vivo*. Protection has been demonstrated in the RV144 human trial but was not associated with *in vitro* neutralization [44]. Volunteers in this trial were at risk for HIV-1 infection by the heterosexual route so they may have been exposed to low doses of virus. Assays with the format outlined in the present study and using virus isolates circulating in the area where the trial was performed may indicate that neutralization can be demonstrated with sera from the trial volunteers. Current SHIV challenge regimes in macaques involve either a bolus [45] with a relatively high dose of virus or repeated low dose challenge until all control macaques are infected [46]. The former has the merit that if protection is seen it is likely to be specific for SHIV; the latter will probably detect an immunization schedule which shows any protection. An alternative protocol would be to titrate virus in immunized and control macaques: the difference in titer would be the measure of protection. Such a protocol could also objectively quantify vaccine efficacy although the number of heterologous virus against which protection can be demonstrated may be too small for this approach to be practicable.

## Materials and Methods

### Ethics statement

The study involved a retrospective analysis of samples from five immunogenicity trials involving 112 adult rhesus macaques, weighing between 4 and 9 kg body and housed at the Biomedical Primate Research Centre (BPRC), The Netherlands. The trials included challenge with SHIV_SF162P4_. The trial protocols were approved (permit numbers DEC#460, DEC#504, DEC#515, DEC#520 and DEC#527) by the Committee on the Ethics of Animal Experiments of Biomedical Primate Research Centre, Animal Welfare Assurance Number VVP/V 9513. The qualification of the members of this committee including their independence from a research institute is requested in the Wet op de Dierproeven (1996). All projects were monitored by a qualified, independent veterinarian, specifically regarding the ethical issues of the projects.

The use of non-human primates in The Netherlands is legalized based on the law: “Wet op de Dierproeven” and adaptations as published in the Staatscourant (48 (1975); 336 (1985); 585 (1992); 435 (1993); 806 (1994); 137(1996); 138(1996); 139 (1996); 5 (1997) and the EU guidelines 86/609/EEG. These laws guarantee the qualification of researchers, veterinary staff and animal caretakers involved in experimental studies and breeding of non-human primates. All animals were either from the breeding stock of the BPRC or purchased from breeding centers in Asia. Identification of imported macaques was confirmed by CITES. The accommodation of laboratory animals was in accordance with animal welfare requirements (1993); Wet op de dierproeven (WOD 1996); Gezondheids-en welzijnswet (GWWD 1996). The animal facilities were licensed to perform studies with genetically modified organisms up to DM3 level (Law on Genetically Modified Organisms, GMO law nr 108, 1996).

All steps were taken to ameliorate the welfare and to avoid the suffering of the animals. At the start of a trial, all animals were in good health and met with the following criteria: no previous immunosuppresive treatment; negative for simian T-lymphotropic virus, simian retrovirus and simian immunodeficiency virus (SIV); low or no IFN-γ, IL2 or IL4 responses against HIV env, gag, pol or nef antigens. They were housed in adjoining, single primate cages, because of the risk of cross-infection following challenge with SHIV. Animals could interact socially with their neighbors and had auditory and visual contact with others in the same room. Enrichment was provided in the form of pieces of wood, mirrors, food puzzles, variety of food and other home made or commercially available enrichment products. The facility was under controlled conditions of humidity (60%), temperature (23–25°C) and lighting (12 hour light/dark cycles). Animals were fed with standard food pellets, fruit and bread. Water was provided *ad libitum*. Animals were sedated with ketamin before blood taking and SHIV challenge. The number of monkeys to be used in individual trials was reduced to a minimum by statistical power calculations and variance values from previous studies to calculate the minimal group sizes to give statistical significance.

At the BPRC all animal handling is performed in the Department of Animal Science (ASD) according to the laws as described above. At the BPRC a large experienced staff is available including full time veterinarians and pathologists. The ASD is regularly inspected by the responsible authorities (VWA) and an independent Animal Welfare Officer.

An outline of the immunization schedules is given in tables 1 and S1. All schedules included an HIV-1_SF162_ envelope glycoprotein immunogen [47], [48], [49], [50]. Macaques in immunogenicity trials one, two and three were immunized in prime boost strategies involving recombinant glycoproteins and synthetic peptides. In trials 4 [51] and 5, the macaques were primed with immunogens in vectors: a replicating adenovirus in trial four and an alphavirus replicon in trial five. All macaques were challenged with SHIV_SF162P4_ grown in rhesus macaque peripheral blood mononuclear cells.

### Virus isolates

Primary HIV-1_SF162_ (original donor: J. Levy [52] and HIV-1_89.6_ (original donor: R. Collman [53]) were obtained from the AIDS Research and Reference Reagent Program, Division of AIDS, NIAID, NIH, Washington DC, USA. The stock was prepared in phytohemagglutinin-transformed, recombinant human IL2 maintained human peripheral blood mononuclear cells (PBMCs). Human PBMCs were donated by volunteers to the Stichting Sanquin Bloedvoorziening, Rotterdam.

### Neutralization assays

All neutralization assays are described as a/b/c where a is the time in hours ( = incubation) during which antibody and virus are incubated prior to exposure to target cells ( = absorption) for b hours. The cells are then washed and incubated for c days ( = culture). The culture phase is timed form the cells' first exposure to virus. All three incubations are at 37 °C. All sera are heat inactivated at 56 °C for one hour.

GHOST(3) Hi-5 cells are human osteosarcoma cells which have been engineered to express the CD4 receptor and green fluorescent protein following infection with HIV-1. The cell line was obtained through the NIH AIDS Research and Reference Reagent Program, Division of AIDS, NIAID, NIH from Dr. Vineet N. KewalRamani and Dr. Dan R. Littman [54]. The cells have been engineered and selected for high expression of CCR5, the co-receptor for the HIV-1 isolates used in this study. The number of individual infectious events can be quantified using a fluorescent activated cell scanner. For GHOST neutralization assays a fixed dilution of each virus stock was chosen based on the results of a previous titration: for neutralization kinetics studies the virus dilution was chosen to give between 200 and 3,000 fluorescent cells per 10,000 recorded events. At higher doses some cells are infected with more than one infectious virus. The dose of virus was adjusted in accordance with the Poisson distribution. One hundred and ninety µls of the fixed virus dilution were incubated for a given interval ( = a hours) with 10 µls of a serum dilution at 37 °C. The virus-antibody mixture was added to GHOST cells which had been seeded 24 h previously at 6×10^4^ cells per well in 24-well cell culture plates [11]. After an absorption period ( = b hours) the cultures were washed three times and cultured for a total of two days ( = c). i. e. the culture period is timed from the first exposure of the cells to the virus. Note that no additives are used to enhance virus binding to target cells. Subsequently, the cells were removed from the plastic by 1 mM EDTA and fixed in formaldehyde at a final concentration of 1%. The cells were analyzed with a FACSsort® flow cytometer (Becton Dickinson). The cells were gated on the basis of forward and side scatter. Using these parameters, uninfected cells were further gated on fluorescence to set the upper limit of the region. The number of infected cells was then determined using the gates with the uninfected cells. The virus titer following incubation with antibody is divided by its titer following incubation as free virus and plotted on a log scale against the incubation (a) or absorption (b) time.

Neutralization kinetics were determined with sera at two dilutions where the ratio was 2.5: 1 in 10 and 1 in 25; 1 in 20 and 1 in 50; 1 in 40 and 1 in 100; 1 in 50 and 1 in 125; 1 in 100 and 1 in 250.

For the standardized and validated neutralization assays the TZM-bl cell line was used [55], [56]. It was obtained through the NIH AIDS Research and Reference Reagent Program, Division of AIDS, NIAID, NIH from Dr. John C. Kappes, Dr. Xiaoyun Wu and Tranzyme Inc. This HeLa cell line is adherent and has been engineered to express CD4 and CCR5 receptors. Following infection with SHIV_SF162P4_ pseudovirus (constructed at the BPRC) the cells produce luciferase, the activity of which can be detected by luminescence. Sera were diluted to give a 1 in 20 dilution and subsequently in a threefold series to a final dilution of 1 in 43,740. Each dilution was mixed with sufficient pseudovirus to give 500,000 counts per second in a Perkin-Elmer (Groningen, The Netherlands) Victor 6016971 luminometer. The mixture includes 15 µg/ml of DEAE and is then incubated for one hour before 10,000 TZM-bl cells are added. The cells are cultured for 48 hours, the supernatants removed and the cells lysed. The cell lysates are transferred to black/white plates, Britelite reagent added and the luciferase activity quantified. Antibody titers are expressed as the dilution of serum required to reduce the luciferase activity in cultures exposed to pseudovirus alone by 50% [26], [27], [28].

### Viral load determinations

For the first, second and third immunization trials the plasma virus load was determined by a quantitative competitive reverse transcription-PCR. Viral RNA was coamplified with a calibrated amount of internal-standard RNA which was added prior to RNA purification. As the target sequence, a highly conserved 267-base pair region in the SIV *gag* gene was chosen. The internal standard was based on the same 267-bp target sequence; however, by PCR, the 26-bp probe region was replaced by a rearranged 26-bp sequence. This fragment was cloned into a transcription vector, and *in vitro* transcripts were synthesized by using T7 RNA polymerase. The RNA was reverse transcribed and amplified within one reaction protocol by rTth DNA polymerase (Perkin-Elmer, Groningen, The Netherlands), using biotinylated primers. The amplification products were alkaline denatured and hybridized in six fivefold dilutions to a capture probe that was covalently bound to microwells. The products were detected by a streptavidin-horseradish peroxidase-mediated calorimetric reaction. The amplified internal standard was hybridized to a different capture probe in separate microwells. The amount of RNA in the plasma sample was determined by calculating the ratio of the optical densities of the sample well and the corresponding internal-standard well. Detection limit is 40 RNA copies/ml [57].

For the fourth and fifth immunization trials SHIV viral loads were determined using an adapted version of a published SIV-gag-based real-time PCR assay [58]. The SIV-probe used was identical to the probe described [58] except that we used the quencher dye Black Hole Quencher 2 instead of TAMRA. The forward (SIV31) and reverse (SIV41) primers were essentially identical to primers SIV.510f and SIV.592r [58], with minor modifications to improve the sensitivity of the assay. The SIV31 and SIV41 primer sequences were 5′-CCAGGATTTCAGGCACTGTC-3′ and 5′-GCTTGATGGTCTCCCACACA-3′, respectively. The PCR was carried out using the Brilliant® QRT-PCR Core Reagent Kit, 1-Step (Stratagene Europe, Amsterdam, The Netherlands) in a 25 µl volume with final concentrations of 160 nM for each primer, 200 nM for the probe, 5.5 nM MgCl_2_, and using 10 µl RNA. RNA was reverse transcribed for 30 min at 45 °C. Then, after a 10 min incubation step at 95 °C, the cDNA was amplified for 40 cycles, consisting of 15 s denaturation at 95 °C, followed by a 1 min annealing-extension step at 60 °C. All the reactions were carried out with an iQ5™ Multicolor Real-Time PCR Detection System (Bio-Rad Laboratories BV, Veenendaal, The Netherlands). Detection limit is 100 RNA copies/ml.

### Statistics

Statistical analyses were performed using GraphPad Prism version 4.00 for Windows, GraphPad Software, San Diego, California, USA, www.graphpad.com. All calculations were performed to four significant figures and then adjusted to three decimal places. Regression coefficients and probability values are given to four significant figures.

Plots of the area under the viral load curve against the neutralizing antibody titer in the TZM-bl assays (Figure 1) were analyzed by linear regression. Regression lines are recorded as y = mx + c where m is the gradient and c the intercept (the value of y when x = 0). Scatter plots were also analyzed by the non-parametric Spearman's rank correlation test. The coefficient of determination (r^2^) gives the proportion of the variability in the dependent variable (in this case the area under the viral load curve, plotted on the vertical, y-axis) which can be attributed to the independent variable (the neutralizing antibody titer, plotted on the horizontal, x-axis). One macaque in the fifth trial was not bled at week 2 and so was excluded from the analysis.

Neutralization rates (Figures 2 and 3): The rate of neutralization with primary isolates of HIV-1 is relatively slow in comparison to other viruses. We chose therefore to present neutralization rates in terms of log_10_ reductions in infectious virus titer per hour rather than the customary log_e_ reductions per second. Plots are presented as the regression line with its 95% confidence band.

Neutralization function comparisons (Figure 4): Data were tested to determine if they followed a normal distribution by the Kolmogorov-Smirnov test, D'Agostino and Pearson omnibus normality test and the Shapiro-Wilk normality test. As many of the samples failed one or other of these tests the data presented in figure 4 are analyzed using the non-parametric Mann-Whitney two-sample test. The ratio data are also presented as linear regression plots in figure 5.

Plots of virus dose against numbers of fluorescent cells were analyzed by linear regression (Table 2 and Figure 6). The gradients of virus incubated with either a control serum or a serum from an immunized macaque were then compared. If there was no significant difference between the gradients, a gradient was calculated from the pooled data and the resulting intercepts of the plots compared.

SHIV_SF162P4_ challenge (see Table S1): immunization strategies were compared using read-outs of peak viral load at week 2 and the areas under the plot of viral load vs time after challenge by a one-way analysis of variance (ANOVAR) or the non-parametric Kruskal-Wallis test. Viral loads at each time point were also analyzed by a two-way ANOVAR. The statistical significance of differences between immunization strategies was determined by Dunnett's Multiple Comparison Test (one-way ANOVAR), Dunn's Multiple Comparison Test (non-parametric test) or Bonferroni post tests (two-way ANOVAR).

## Supporting Information

Table S1 Outline of immunization schedules for five rhesus macaque immunogenicity and SHIV_SF162P4_ challenge studies.
(DOC)

## References

[1] Kramer VG, Siddappa NB, Ruprecht RM (2007). Passive immunization as tool to identify protective HIV-1 Env epitopes.. Current HIV research.

[2] Haigwood NL (2004). Predictive value of primate models for AIDS.. AIDS reviews.

[3] Shedlock DJ, Silvestri G, Weiner DB (2009). Monkeying around with HIV vaccines: using rhesus macaques to define ‘gatekeepers’ for clinical trials.. Nature reviews Immunology.

[4] Luciw PA, Pratt-Lowe E, Shaw KE, Levy JA, Cheng-Mayer C (1995). Persistent infection of rhesus macaques with T-cell-line-tropic and macrophage-tropic clones of simian/human immunodeficiency viruses (SHIV).. Proceedings of the National Academy of Sciences of the United States of America.

[5] Harouse JM, Gettie A, Tan RC, Blanchard J, Cheng-Mayer C (1999). Distinct pathogenic sequela in rhesus macaques infected with CCR5 or CXCR4 utilizing SHIVs.. Science.

[6] Parren PW, Marx PA, Hessell AJ, Luckay A, Harouse J (2001). Antibody protects macaques against vaginal challenge with a pathogenic R5 simian/human immunodeficiency virus at serum levels giving complete neutralization in vitro.. Journal of virology.

[7] Barnett SW, Burke B, Sun Y, Kan E, Legg H (2010). Antibody-mediated protection against mucosal simian-human immunodeficiency virus challenge of macaques immunized with alphavirus replicon particles and boosted with trimeric envelope glycoprotein in MF59 adjuvant.. Journal of virology.

[8] Heeney JL, Teeuwsen VJ, van Gils M, Bogers WM, De Giuli Morghen C (1998). beta-chemokines and neutralizing antibody titers correlate with sterilizing immunity generated in HIV-1 vaccinated macaques.. Proceedings of the National Academy of Sciences of the United States of America.

[9] Davis D, Donners H, Willems B, Lovgren-Bengtsson K, Akerblom L (2004). Neutralization of primary HIV-1 SF13 can be detected in extended incubation phase assays with sera from monkeys immunized with recombinant HIV-1 SF2 gp120.. Vaccine.

[10] Davis D, Donners H, Willems B, Ntemgwa M, Vermoesen T (2006). Neutralization kinetics of sensitive and resistant subtype B primary human immunodeficiency virus type 1 isolates.. Journal of medical virology.

[11] Cecilia D, KewalRamani VN, O'Leary J, Volsky B, Nyambi P (1998). Neutralization profiles of primary human immunodeficiency virus type 1 isolates in the context of coreceptor usage.. Journal of virology.

[12] Mascola JR (2003). Defining the protective antibody response for HIV-1.. Current molecular medicine.

[13] Polonis VR, Brown BK, Rosa Borges A, Zolla-Pazner S, Dimitrov DS (2008). Recent advances in the characterization of HIV-1 neutralization assays for standardized evaluation of the antibody response to infection and vaccination.. Virology.

[14] Plotkin SA (2008). Vaccines: correlates of vaccine-induced immunity.. Clinical infectious diseases : an official publication of the Infectious Diseases Society of America.

[15] Robbins JB, Schneerson R, Szu SC (1996). Hypothesis: how licensed vaccines confer protective immunity.. Advances in experimental medicine and biology.

[16] Dimmock NJ (1993). Neutralization of animal viruses.. Current topics in microbiology and immunology.

[17] Donners H, Davis D, Willems B, van der Groen G (2003). Inter-subtype cross-neutralizing antibodies recognize epitopes on cell-associated HIV-1 virions.. Journal of medical virology.

[18] Davis D, Donners H, Willems B, Vermoesen T, Heyndrickx L (2003). Epitopes corresponding to the envelope genetic subtype are present on the surface of free virions of HIV-1 group M primary isolates and can be detected in neutralization assays with extended incubation phases.. Journal of medical virology.

[19] Polonis VR, Schuitemaker H, Bunnik EM, Brown BK, Scarlatti G (2009). Impact of host cell variation on the neutralization of HIV-1 in vitro.. Current opinion in HIV and AIDS.

[20] Mann AM, Rusert P, Berlinger L, Kuster H, Gunthard HF (2009). HIV sensitivity to neutralization is determined by target and virus producer cell properties.. AIDS.

[21] Fenyo EM, Heath A, Dispinseri S, Holmes H, Lusso P (2009). International network for comparison of HIV neutralization assays: the NeutNet report.. PloS one.

[22] Zolla-Pazner S, Sharpe S (1995). A resting cell assay for improved detection of antibody-mediated neutralization of HIV type 1 primary isolates.. AIDS research and human retroviruses.

[23] Holl V, Peressin M, Decoville T, Schmidt S, Zolla-Pazner S (2006). Nonneutralizing antibodies are able to inhibit human immunodeficiency virus type 1 replication in macrophages and immature dendritic cells.. Journal of virology.

[24] Choudhry V, Zhang MY, Harris I, Sidorov IA, Vu B (2006). Increased efficacy of HIV-1 neutralization by antibodies at low CCR5 surface concentration.. Biochemical and biophysical research communications.

[25] Davis D, Trischmann H, Stephens DM, Lachmann PJ (2001). Antibodies raised to short synthetic peptides with sequences derived from HIV-1 SF2 gp120 can both neutralize and enhance HIV-1 SF13: a later variant isolated from the same host.. Journal of medical virology.

[26] Li M, Gao F, Mascola JR, Stamatatos L, Polonis VR (2005). Human immunodeficiency virus type 1 env clones from acute and early subtype B infections for standardized assessments of vaccine-elicited neutralizing antibodies.. Journal of virology.

[27] Montefiori DC (2009). Measuring HIV neutralization in a luciferase reporter gene assay.. Methods in molecular biology.

[28] Seaman MS, Janes H, Hawkins N, Grandpre LE, Devoy C (2010). Tiered categorization of a diverse panel of HIV-1 Env pseudoviruses for assessment of neutralizing antibodies.. Journal of virology.

[29] Seaman MS, Leblanc DF, Grandpre LE, Bartman MT, Montefiori DC (2007). Standardized assessment of NAb responses elicited in rhesus monkeys immunized with single- or multi-clade HIV-1 envelope immunogens.. Virology.

[30] Zhuge W, Jia F, Adany I, Narayan O, Stephens EB (1997). Plasmas from lymphocyte- and macrophage-tropic SIVmac-infected macaques have antibodies with a broader spectrum of virus neutralization activity in macrophage versus lymphocyte cultures.. Virology.

[31] Layne SP, Dembo M (1992). The auto-regulation model: a unified concept of how HIV regulates its infectivity, pathogenesis and persistence.. International reviews of immunology.

[32] Mascola JR, Snyder SW, Weislow OS, Belay SM, Belshe RB (1996). Immunization with envelope subunit vaccine products elicits neutralizing antibodies against laboratory-adapted but not primary isolates of human immunodeficiency virus type 1. The National Institute of Allergy and Infectious Diseases AIDS Vaccine Evaluation Group.. The Journal of infectious diseases.

[33] Myszka DG, Sweet RW, Hensley P, Brigham-Burke M, Kwong PD (2000). Energetics of the HIV gp120-CD4 binding reaction.. Proceedings of the National Academy of Sciences of the United States of America.

[34] Wyatt R, Sodroski J (1998). The HIV-1 envelope glycoproteins: fusogens, antigens, and immunogens.. Science.

[35] Keele BF, Giorgi EE, Salazar-Gonzalez JF, Decker JM, Pham KT (2008). Identification and characterization of transmitted and early founder virus envelopes in primary HIV-1 infection.. Proceedings of the National Academy of Sciences of the United States of America.

[36] Abrahams MR, Anderson JA, Giorgi EE, Seoighe C, Mlisana K (2009). Quantitating the multiplicity of infection with human immunodeficiency virus type 1 subtype C reveals a non-poisson distribution of transmitted variants.. Journal of virology.

[37] Li H, Bar KJ, Wang S, Decker JM, Chen Y (2010). High Multiplicity Infection by HIV-1 in Men Who Have Sex with Men.. PLoS pathogens.

[38] Bar KJ, Li H, Chamberland A, Tremblay C, Routy JP (2010). Wide variation in the multiplicity of HIV-1 infection among injection drug users.. Journal of virology.

[39] Russell ES, Kwiek JJ, Keys J, Barton K, Mwapasa V (2011). The Genetic Bottleneck in Vertical Transmission of Subtype C HIV-1 Is Not Driven by Selection of Especially Neutralization-Resistant Virus from the Maternal Viral Population.. Journal of virology.

[40] Zhang H, Rola M, West JT, Tully DC, Kubis P (2010). Functional properties of the HIV-1 subtype C envelope glycoprotein associated with mother-to-child transmission.. Virology.

[41] Derdeyn CA, Decker JM, Bibollet-Ruche F, Mokili JL, Muldoon M (2004). Envelope-constrained neutralization-sensitive HIV-1 after heterosexual transmission.. Science.

[42] Chohan B, Lang D, Sagar M, Korber B, Lavreys L (2005). Selection for human immunodeficiency virus type 1 envelope glycosylation variants with shorter V1-V2 loop sequences occurs during transmission of certain genetic subtypes and may impact viral RNA levels.. Journal of virology.

[43] Frost SD, Liu Y, Pond SL, Chappey C, Wrin T (2005). Characterization of human immunodeficiency virus type 1 (HIV-1) envelope variation and neutralizing antibody responses during transmission of HIV-1 subtype B.. Journal of virology.

[44] Rerks-Ngarm S, Pitisuttithum P, Nitayaphan S, Kaewkungwal J, Chiu J (2009). Vaccination with ALVAC and AIDSVAX to prevent HIV-1 infection in Thailand.. The New England journal of medicine.

[45] Nishimura Y, Igarashi T, Haigwood N, Sadjadpour R, Plishka RJ (2002). Determination of a statistically valid neutralization titer in plasma that confers protection against simian-human immunodeficiency virus challenge following passive transfer of high-titered neutralizing antibodies.. Journal of virology.

[46] Hessell AJ, Poignard P, Hunter M, Hangartner L, Tehrani DM (2009). Effective, low-titer antibody protection against low-dose repeated mucosal SHIV challenge in macaques.. Nature medicine.

[47] Demberg T, Florese RH, Heath MJ, Larsen K, Kalisz I (2007). A replication-competent adenovirus-human immunodeficiency virus (Ad-HIV) tat and Ad-HIV env priming/Tat and envelope protein boosting regimen elicits enhanced protective efficacy against simian/human immunodeficiency virus SHIV89.6P challenge in rhesus macaques.. Journal of virology.

[48] Srivastava IK, Stamatatos L, Kan E, Vajdy M, Lian Y (2003). Purification, characterization, and immunogenicity of a soluble trimeric envelope protein containing a partial deletion of the V2 loop derived from SF162, an R5-tropic human immunodeficiency virus type 1 isolate.. Journal of virology.

[49] Stamatatos L, Lim M, Cheng-Mayer C (2000). Generation and structural analysis of soluble oligomeric gp140 envelope proteins derived from neutralization-resistant and neutralization-susceptible primary HIV type 1 isolates.. AIDS research and human retroviruses.

[50] Xu R, Srivastava IK, Greer CE, Zarkikh I, Kraft Z (2006). Characterization of immune responses elicited in macaques immunized sequentially with chimeric VEE/SIN alphavirus replicon particles expressing SIVGag and/or HIVEnv and with recombinant HIVgp140Env protein.. AIDS research and human retroviruses.

[51] Bogers WM, Davis D, Baak I, Kan E, Hofman S (2008). Systemic neutralizing antibodies induced by long interval mucosally primed systemically boosted immunization correlate with protection from mucosal SHIV challenge.. Virology.

[52] Cheng-Mayer C, Levy JA (1988). Distinct biological and serological properties of human immunodeficiency viruses from the brain.. Annals of neurology.

[53] Collman R, Balliet JW, Gregory SA, Friedman H, Kolson DL (1992). An infectious molecular clone of an unusual macrophage-tropic and highly cytopathic strain of human immunodeficiency virus type 1.. Journal of virology.

[54] Morner A, Bjorndal A, Albert J, Kewalramani VN, Littman DR (1999). Primary human immunodeficiency virus type 2 (HIV-2) isolates, like HIV-1 isolates, frequently use CCR5 but show promiscuity in coreceptor usage.. Journal of virology.

[55] Wei X, Decker JM, Liu H, Zhang Z, Arani RB (2002). Emergence of resistant human immunodeficiency virus type 1 in patients receiving fusion inhibitor (T-20) monotherapy.. Antimicrobial agents and chemotherapy.

[56] Derdeyn CA, Decker JM, Sfakianos JN, Wu X, O'Brien WA (2000). Sensitivity of human immunodeficiency virus type 1 to the fusion inhibitor T-20 is modulated by coreceptor specificity defined by the V3 loop of gp120.. Journal of virology.

[57] Verschoor EJ, Mooij P, Oostermeijer H, van der Kolk M, ten Haaft P (1999). Comparison of immunity generated by nucleic acid-, MF59-, and ISCOM-formulated human immunodeficiency virus type 1 vaccines in Rhesus macaques: evidence for viral clearance.. Journal of virology.

[58] Leutenegger CM, Higgins J, Matthews TB, Tarantal AF, Luciw PA (2001). Real-time TaqMan PCR as a specific and more sensitive alternative to the branched-chain DNA assay for quantitation of simian immunodeficiency virus RNA.. AIDS research and human retroviruses.

