# Supplementary material for: Protection in Macaques Immunized with HIV-1 Candidate Vaccines Can Be Predicted Using the Kinetics of Their Neutralizing Antibodies
Source: PLoS One. 2011 Dec 28;6(12):e28974. doi: 10.1371/journal.pone.0028974 (PMC3247218; doi:10.1371/journal.pone.0028974)
Supplement: Table S1 — Outline of immunization schedules for five rhesus macaque immunogenicity and SHIVSF162P4 challenge studies. (DOC) [file pone.0028974.s001.doc]

Supplementary Table 1

Outline of immunization schedules for five rhesus macaque immunogenicity and SHIV _SF162P4_ challenge studies.

First pre-clinical immunization trial

Twenty imported macaques in two replicates of 10 allocated into four immunization groups of 4 plus four control macaques.

Schedule: Weeks 0, 6, 16, 36 and 47.

Immunizations at weeks 0, 6 and 16 in ISCOM matrix ;

Subsequent immunizations in MF59 + CpG

Group 1.1: Recombinant HIV-1 SF162 ΔV2 gp140 (subtype B) for all five immunizations.

(Macaques Ri 424, Ri 428, Ri 358 & Ri 395)

Group 1.2: Primed with recombinant HIV-1 SF162 ΔV2 gp140 at weeks 0 and 6

Boosted with synthetic peptide homologous V3 linear epitope (NNNT RKSITIGPGRAC) and mimotopes to monoclonal antibodies 19b (RGRKLESSWYNTVWRC) and IgG1 b12 (NWPRWWEEFVD KHSSC) covalently coupled to Keyhole Limpet Haemocyanin (KLH) at weeks 6, 16, 37 and 46.

(Macaques Ri 232, Ri 340, Ri 305 & Ri 316)

Group 1.3: Recombinant HIV-1 TV1 ΔV2 gp140 (subtype C) for all five immunizations.

(Macaques 97046, Ri 324, Ri 141 & Ri 197)

Group 1.4: Primed with recombinant HIV-1 TV1 ΔV2 gp140 at weeks 0 and 6

Boosted with synthetic peptide homologous V3 linear epitope (NNNT RKSVRIGPGQAC) and mimotopes to monoclonal antibodies 19b (RGRKLESSWYNTVWRC) and IgG1 b12 (NWPRWWEEFVD KHSSC) covalently coupled to KLH at weeks 6, 16, 37 and 46.

(Macaques Ri 353, Ri 389, Ri 434 & Ri 435)

Group 1.5 Controls.

(Macaques Ri 323, Ri 269, Ri 327 & 97061)

Intravenous challenge at week 55 with 50 TCID_50_ of first DAIDS commissioned stock of SHIV_SF162P4_  .

Two-way Analysis of Variance, viral loads vs time, weeks 2 – 12 inclusive.

Source of Variation Df Sum-of-squares Mean square F Significance

Interaction 16 5.143 0.3214 0.8923 0.5807

Time 4 110.6 27.65 76.77 <0.0001

Immunization schedule 4 10.73 2.683 2.762 0.0667

Subjects (matching) 15 14.57 0.9715 2.697 0.0033

Residual 60 21.61 0.3602

Bonferroni post tests:

Recombinant HIV-1 SF162 vs Controls at week 2: p < 0.05

HIV-1 SF162 Rec Prime, Pep Boost vs Controls at week 4: p < 0.05

All other comparisons vs controls: p > 0.05

One-way Analysis of Variance, viral loads at week 2:

ANOVA Table

Source of Variation Sum-of-squares Df Mean square F Significance

Treatment (between columns) 5.392 4 1.348 2.015 0.1440

Residual (within columns) 10.04 15 0.6690

Total 15.43 19

Dunnett's Multiple Comparison Test

No immunization schedule is significantly different from the controls.

Kruskal-Wallis test

H = 7.459 p = 0.1135

Dunn's Multiple Comparison Test

No immunization schedule is significantly different from the controls.

One-way Analysis of Variance, area under viral load curve up to week 8:

Source of Variation Sum-of-squares Df Mean square F Significance Treatment (between columns) 160.2 4 40.05 2.416 0.0946

Residual (within columns) 248.7 15 16.58

Total 408.9 19

Dunnett's Multiple Comparison Test

No immunization schedule is significantly different from the controls.

Kruskal-Wallis test

H = 6.343 p = 0.1750

Dunn's Multiple Comparison Test

No immunization schedule is significantly different from the controls.

Second pre-clinical immunization trial

20 BPRC-bred macaques in a SINGLE replicate randomised into three immunization groups of 5 plus five control macaques.

Schedule: Weeks 0, 6 and 16.

All immunizations in MF59 + CpG

Group 2.1: Recombinant HIV-1 461 gp140 (subtype A), HIV-1 SF162 gp140 (subtype B) and HIV-1 TV1 gp140 (subtype C) for all three immunizations.

(Macaques 95067, 96032, R 99023, R 00058 & BB 203)

Group 2.2: Priming with recombinant HIV-1 461 gp140 (subtype A), HIV-1 SF162 gp140 (subtype B) and HIV-1 TV1 gp140 (subtype C) for immunization at weeks 0 and 6; Boosting with HIV-1 SF162 cyclised V3 peptide (CTRPNNNTRKSITIGPGRAFYATGDIIGDIRQAHC) and also synthetic peptides to HIV-1 SF162 V2 (IRNKMQKEYA LFYKLC) linear epitope and mimotope to monoclonal antibody IgG1 b12 (NWPRWWEEFVD KHSSC) both covalently coupled to KLH, at weeks 6 and 16.

(Macaques 8944, 96064, R 00055, BB 206 & R 00010)

Group 2.3: Priming with recombinant HIV-1 461 gp140 (subtype A), HIV-1 SF162 gp140 (subtype B) and HIV-1 TV1 gp140 (subtype C) for immunization at weeks 0 and 6; Boosting with HIV-1 TV1 cyclised V3 peptide (CTRPNNNTRKSVRIGPGQAFYATNDVIGNIRQAHC) and also synthetic peptides to linear peptide binding to monoclonal antibody 2F5 (NEQELLELDKWASLWNC) and mimotope to monoclonal antibody IgG1 b12 (HERSYMFSDLENRC) , both covalently coupled to KLH, at weeks 6 and 16.

(Macaques C 67, 95051, R 00011, R 99012 & BB 201)

Group 2.4 Controls.

(Macaques 9302, 97024, R 00030, R 01011 & BB 205)

Intrarectal challenge at week 24 with 1,800 TCID_50_ of second DAIDS commissioned stock of SHIV_SF162P4_ .

Two-way Analysis of Variance, viral loads vs time, weeks 2 – 12 inclusive.

Source of Variation Df Sum-of-squares Mean square F Significance

Interaction 12 13.56 1.130 2.588 0.0072

Time 4 50.35 12.59 28.82 <0.0001

Immunization schedule 3 9.637 3.212 2.896 0.0675

Subjects (matching) 16 17.75 1.109 2.540 0.0044

Residual 64 27.95 0.4367

Bonferroni post tests:

Recombinant alone vs Controls at week 4: p < 0.001

Recombinant prime, HIV-1 SF162 boost vs Controls at week 4: p < 0.001

Recombinant prime, HIV-1 TV1 boost vs Controls at week 4: p < 0.05

One-way Analysis of Variance, viral loads at week 2:

ANOVA Table

Source of Variation Sum-of-squares Df Mean square F Significance

Treatment (between columns) 4.764 3 1.588 0.8451 0.4891

Residual (within columns) 30.06 16 1.879

Total 34.8 3 19

Dunnett's Multiple Comparison Test

No immunization schedule is significantly different from the controls.

Kruskal-Wallis test

H = 2.457 p = 0.4830

Dunn's Multiple Comparison Test

No immunization schedule is significantly different from the controls.

One-way Analysis of Variance, areas from week 0 – week8:

ANOVA Table

Source of Variation Sum-of-squares Df Mean square F Significance

Treatment (between columns) 245.7 3 81.89 4.451 0.0187

Residual (within columns) 294.3 16 18.40

Total 540.0 19

Dunnett's Multiple Comparison Test

No immunization schedule is significantly different from the controls.

Kruskal-Wallis test

H = 9.738 p = 0.0209

Dunn's Multiple Comparison Test

Recombinant alone vs Controls: p < 0.05

Recombinant prime, HIV-1 SF162 boost vs Controls: p < 0.05

Recombinant prime, HIV-1 TV1 boost vs Controls: p > 0.05 (Not significant)

Third pre-clinical immunization trial

20 BPRC-bred macaques in a SINGLE replicate randomised into three immunization groups of 5 plus five control macaques.

Schedule: Weeks 0, 6 and 16.

All immunizations in MF59 + CpG

Group 3.1: Recombinant HIV-1 461 gp140 (subtype A), HIV-1 SF162 gp140 (subtype B) and HIV-1 TV1 gp140 (subtype C) for all three immunizations.

(Macaques R 01090, R 00040, R 01089, R 01093 & R 99008)

Group 3.2: Priming with synthetic HIV-1 SF162 cyclised V3 peptide (CTRPNNN TRKSITIGPGRAFYATGDIIGDIRQAHC) and also synthetic peptides to HIV-1 SF162 V2 linear epitope (IRNKMQKEYA LFYKLC) and mimotope to monoclonal antibody IgG1 b12 (NWPRWWEEFVD KHSSC) , both covalently coupled to KLH, at weeks 0 and 6; Boosting with recombinant HIV-1 461 gp140 (subtype A), HIV-1 SF162 gp140 (subtype B) and HIV-1 TV1 gp140 (subtype C) for immunization at weeks 6 and 16.

(Macaques R 01012, 8813, R 01070, 8865, & R 99037)

Group 3.3: Priming with synthetic HIV-1 TV1 cyclised V3 peptide (CTRPNNNT RKSVRIGPGQAFYATNDVIGNIRQAHC) as well as synthetic peptides to linear sequence which binds monoclonal antibody 2F5 (NEQELLELDKWASLWNC) and mimotope to monoclonal antibody IgG1 b12 (HERSYMFSDLENRC) both covalently coupled to KLH, at weeks 0 and 6; Boosting with recombinant HIV-1 461 gp140 (subtype A), HIV-1 SF162 gp140 (subtype B) and HIV-1 TV1 gp140 (subtype C) for immunization at weeks 6 and 16.

(Macaques R 00042, R 00057, BB204, 98041 & 8758)

Group 3.4: Controls

(Macaques R 99006, 96050, R00056, R 99004 & 96015)

Intrarectal challenge at week 24 with 1,800 TCID_50_ of second DAIDS commissioned stock of SHIV_SF162P4_ .

Two-way Analysis of Variance, viral loads vs time, weeks 2 – 12 inclusive.

Source of Variation Df Sum-of-squares Mean square F Significance

Interaction 12 12.22 1.018 1.322 0.2286

Time 4 31.18 7.796 10.12 <0.0001

Immunization schedule 3 31.99 10.66 2.831 0.0715

Subjects (matching) 16 60.25 3.766 4.888 <0.0001

Residual 64 49.31 0.7704

Bonferroni post tests:

Recombinant alone vs Controls at week 2: p < 0.01

Recombinant prime, HIV-1 SF162 boost vs Controls at week 2: p < 0.01

Recombinant prime, HIV-1 TV1 boost vs Controls: p > 0.05 (N. S.) at any time point

One-way Analysis of Variance, viral loads at week 2:

ANOVA Table

Source of Variation Sum-of-squares Df Mean square F Significance

Treatment (between columns) 16.67 3 5.557 3.051 0.0589

Residual (within columns) 29.15 16 1.822

Total 45.82 19

Dunnett's Multiple Comparison Test

Recombinant alone vs controls: p < 0.05

Kruskal-Wallis test

H = 8.803 p = 0.0320

Dunn's Multiple Comparison Test

Recombinant alone vs controls: p < 0.05

One-way Analysis of Variance, areas from week 0 – week8 (Exclude control 96015):

ANOVA Table

Source of Variation Sum-of-squares Df Mean square F Significance

Treatment (between columns) 172.0 3 57.35 3.778 0.0335

Residual (within columns) 227.7 15 15.18

Total 399.7 18

Dunnett's Multiple Comparison Test

HIV-1 SF162 peptide prime vs control: p < 0.05

Kruskal-Wallis test

H = 8.472 p = 0.0372

Dunn's Multiple Comparison Test

HIV-1 SF162 peptide prime vs control: p < 0.01

Fourth pre-clinical immunization trial

Twelve imported macaques in a SINGLE replicate allocated into two immunization groups of 4 plus four control macaques.

Schedule: Weeks 0, 12, 24 and 36.

Week 0 and 12: Priming with replicating Adenovirus vector (Ad5hr-HIV89.6PΔCFI gp140) : intranasal at week 0 and intratracheal at week 12;

Weeks 24 and 36: Boosting with glycoproteins in MF59 + CpG or recombinant VEE/SIN (Venezuelan equine encephalitis /Sindbis) virus replicon vector .

Group 4.1: Primed with adenovirus vector; boosted with oligomeric recombinant HIV-1 SF162 gp140 as glycoprotein.

(Macaques Ri 372, Ri 101, Ri 111, Ri108)

Group 4.2: Primed with adenovirus vector; boosted with recombinant VEE replicon vector particles encoding oligomeric HIV-1 SF162 ΔV2 gp140.

(Macaques Ri 377, Ri 103, Ri 115, Ri 119)

Group 4.3: Controls.

(Macaques Ri 379, Ri 104, Ri 130, Ri 118)

Intrarectal challenge at week 44 with 1,800 TCID_50_ of second DAIDS commissioned stock of SHIV_SF162P4_ .

Two-way Analysis of Variance, viral loads vs time, weeks 1 – 12 inclusive.

Source of Variation Df Sum-of-squares Mean square F Significance

Interaction 8 10.17 1.272 2.028 0.0708

Time 4 31.17 7.791 12.42 <0.0001

Immunization schedule 2 24.50 12.25 11.20 0.0036

Subjects (matching) 9 9.846 1.094 1.744 0.1144

Residual 36 22.58 0.6271

Bonferroni post tests:

Boosted with HIV-1 SF162 vs Controls at week 2: p < 0.001; week 4: p < 0.01

Boosted with replicons vs Controls at week 2: p < 0.01; week 4: p < 0.01

One-way Analysis of Variance, viral loads at week 2:

ANOVA Table

Source of Variation Sum-of-squares Df Mean square F Significance

Treatment (between columns) 19.41 2 9.704 3.600 0.0710

Residual (within columns) 24.26 9 2.695

Total 43.67 11

Dunnett's Multiple Comparison Test

Neither immunization schedule is significantly different from controls.

Kruskal-Wallis test

H = 4.011 p = 0.1346

Dunn's Multiple Comparison Test

Neither immunization schedule is significantly different from controls.

One-way Analysis of Variance, areas from week 0 – week8:

ANOVA Table

Source of Variation Sum-of-squares Df Mean square F Significance

Treatment (between columns) 395.3 2 197.6 13.62 0.0019

Residual (within columns) 130.6 9 14.51

Total 525.9 11

Dunnett's Multiple Comparison Test

Boosted with HIV-1 SF162 vs Controls: p < 0.01

Boosted with replicons vs Controls: p < 0.01

Kruskal-Wallis test

H = 8.312 p = 0.0157

Dunn's Multiple Comparison Test

Boosted with HIV-1 SF162 vs Controls: p < 0.05

Fifth pre-clinical immunization trial

Forty imported macaques in TWO REPLICATES of twenty allocated into four immunization groups of 8 plus eight control macaques.

Schedule: Weeks 0, 4, 12, 24 and 36.

Priming at weeks 0, 4 and 12 with recombinant VEE/SIN (Venezuelan equine encephalitis /Sindbis) virus replicon vector particles or empty VEE replicons;

Boosting at weeks 24 and 36 with glycoproteins in MF59 plus CpG .

Group 5.1: Primed with recombinant VEE virus replicons encoding oligomeric HIV-1 SF162 ΔV2 gp140 (= subtype B); boosting with oligomeric recombinant HIV-1 SF162 gp140 (= subtype B) as glycoprotein.

(Macaques Ri 450, Ri 295, Ri 371, Ri 392, C 019, Ri 416, Ri 4188 & Ri 12101)

Group 5.2: Primed with recombinant VEE replicons encoding oligomeric HIV-1 MJ4 gp140 (= subtype C); boosting with oligomeric recombinant HIV-1 MJ4 gp140 (= subtype C) as glycoprotein.

(Macaques C 035, Ri 105, Ri 297, Ri 390, Ri 384, Ri 470, Ri 10282 & Ri 12195)

Group 5.3: Primed with recombinant VEE replicons encoding oligomericHIV-1 SF162 ΔV2 gp140 (= subtype B) plus recombinant VEE replicons encoding oligomeric HIV-1 MJ4 gp140 (= subtype C); boosting with oligomeric recombinant HIV-1 SF162 gp140 (= subtype B) plus oligomeric recombinant HIV-1 MJ4 gp140 (= subtype C) as glycoproteins.

(Macaques Ri 485, Ri 086, Ri 113, Ri 441, C 109, Ri 469, Ri 3242 & Ri 12006)

Group 5.4: Primed with empty VEE replicons; boosting with oligomeric recombinant HIV-1 SF162 gp140 (= subtype B) plus oligomeric recombinant HIV-1 MJ4 gp140 (= subtype C) as glycoproteins.

(Macaques C 121, Ri 090, Ri 325, Ri 383, Ri 415, Ri 462, Ri 11452 & Ri 12222)

Group 5.5: Controls.

(Macaques C 138, Ri 486, Ri 513, Ri 12246, Ri 304, Ri 337, Ri 476 & Ri 11035)

Intrarectal challenge at week 44 with log_10_ 2.1 MID_50_ of BPRC prepared stock of SHIV_SF162P4_ .

Two-way Analysis of Variance, viral loads vs time, weeks 1 – 12 inclusive.*

Source of Variation Df Sum-of-squares Mean square F Significance

Interaction 16 38.66 2.417 3.717 <0.0001

Time 4 214.8 53.70 82.61 <0.0001

Immunization schedule 4 44.73 11.18 9.687 <0.0001

Subjects (matching) 35 40.41 1.154 1.776 0.0104

Residual 140 91.01 0.6501

* Ri 441 (Group 5.3) weeks 1 and 2 values missing: replaced by mean of other 7 macaques from same treatment at same time points;

C138 (Control) weeks 1 and 2 values missing: replaced by mean of other 7 control macaques at same time points;

C109 (Group 5.3) week 1 value missing but negative until week 8 becoming positive at week 12: replaced with background viral load of log_10_ 2.00

Bonferroni post tests:

Primed and boosted with HIV-1 SF162 vs Controls at week 1: p < 0.01; week 2: p < 0.001; week4: p < 0.001.

Primed and boosted with HIV-1 MJ4 vs Controls: no significant protection at any time point.

Primed and boosted with both HIV-1 SF162 and HIV-1 MJ4 vs Controls at week 1: p < 0.01; week 2: p < 0.001; week4: p < 0.001.

Primed with empty replicons and boosted with both HIV-1 SF162 and HIV-1 MJ4 recombinant gp140 vs Controls at week 1: p < 0.001; week 2: p > 0.05 (Not Significant; week4: p < 0.001.

One-way Analysis of Variance, viral loads at week 2:

ANOVA Table

Source of Variation Sum-of-squares Df Mean square F Significance

Treatment (between columns) 44.85 4 11.21 3.414 0.0192

Residual (within columns) 108.4 33 3.285

Total 153.3 37

Dunnett's Multiple Comparison Test

Both prime, boost immunization schedules which include HIV-1 SF162 immunogens (Group 5.1 and Group 5.3) induce statistically significant reductions in viral load when compared to control macaques: p < 0.05 for both.

Kruskal-Wallis test

H = 14.00 p = 0.0073

Dunn's Multiple Comparison Test

Both prime, boost immunization schedules which include HIV-1 SF162 immunogens (Group 5.1 and Group 5.3) induce statistically significant reductions in viral load when compared to control macaques: p < 0.01 for both.

One-way Analysis of Variance, areas from week 0 – week8:

ANOVA Table

Source of Variation Sum-of-squares Df Mean square F Significance

Treatment (between columns) 643.7 4 160.9 11.99 <0.0001

Residual (within columns) 469.8 35 13.42

Total 1113 39

Dunnett's Multiple Comparison Test

Both prime, boost immunization schedules which include HIV-1 SF162 immunogens (Group 5.1 and Group 5.3) induce statistically significant reductions in viral load when compared to control macaques: p < 0.01 for both.

Also, macaques which have only been boosted with HIV-1 SF162 recombinant gp140 and HIV-1 MJ4 (Group 5.4) have a statistically significant reduction in viral load when compared to control macaques: p < 0.01

Kruskal-Wallis test

H = 21.42 p = 0.0003

Dunn's Multiple Comparison Test

Both prime, boost immunization schedules which include HIV-1 SF162 immunogens (Group 5.1 and Group 5.3) induce statistically significant reductions in viral load when compared to control macaques: p < 0.01 for boosting with HIV-1 SF162 recombinant gp140; p < 0.001 for boosting with mixture of HIV-1 SF162 and HIV-1 MJ4 recombinant gp140.

References

1. Lovgren Bengtsson K, Morein B, Osterhaus AD (2011) ISCOM technology-based Matrix M adjuvant: success in future vaccines relies on formulation. Expert review of vaccines 10: 401-403.

2. Mosca F, Tritto E, Muzzi A, Monaci E, Bagnoli F, et al. (2008) Molecular and cellular signatures of human vaccine adjuvants. Proceedings of the National Academy of Sciences of the United States of America 105: 10501-10506.

3. Srivastava IK, Stamatatos L, Kan E, Vajdy M, Lian Y, et al. (2003) Purification, characterization, and immunogenicity of a soluble trimeric envelope protein containing a partial deletion of the V2 loop derived from SF162, an R5-tropic human immunodeficiency virus type 1 isolate. Journal of virology 77: 11244-11259.

4. Stamatatos L, Lim M, Cheng-Mayer C (2000) Generation and structural analysis of soluble oligomeric gp140 envelope proteins derived from neutralization-resistant and neutralization-susceptible primary HIV type 1 isolates. AIDS research and human retroviruses 16: 981-994.

5. Boots LJ, McKenna PM, Arnold BA, Keller PM, Gorny MK, et al. (1997) Anti-human immunodeficiency virus type 1 human monoclonal antibodies that bind discontinuous epitopes in the viral glycoproteins can identify mimotopes from recombinant phage peptide display libraries. AIDS research and human retroviruses 13: 1549-1559.

6. Harouse JM, Gettie A, Tan RC, Blanchard J, Cheng-Mayer C (1999) Distinct pathogenic sequela in rhesus macaques infected with CCR5 or CXCR4 utilizing SHIVs. Science 284: 816-819.

7. Luciw PA, Pratt-Lowe E, Shaw KE, Levy JA, Cheng-Mayer C (1995) Persistent infection of rhesus macaques with T-cell-line-tropic and macrophage-tropic clones of simian/human immunodeficiency viruses (SHIV). Proceedings of the National Academy of Sciences of the United States of America 92: 7490-7494.

8. Lian Y, Srivastava I, Gomez-Roman VR, Zur Megede J, Sun Y, et al. (2005) Evaluation of envelope vaccines derived from the South African subtype C human immunodeficiency virus type 1 TV1 strain. Journal of virology 79: 13338-13349.

9. Parker CE, Deterding LJ, Hager-Braun C, Binley JM, Schulke N, et al. (2001) Fine definition of the epitope on the gp41 glycoprotein of human immunodeficiency virus type 1 for the neutralizing monoclonal antibody 2F5. Journal of virology 75: 10906-10911.

10. Zwick MB, Bonnycastle LL, Menendez A, Irving MB, Barbas CF, 3rd, et al. (2001) Identification and characterization of a peptide that specifically binds the human, broadly neutralizing anti-human immunodeficiency virus type 1 antibody b12. Journal of virology 75: 6692-6699.

11. Polacino P, Larsen K, Galmin L, Suschak J, Kraft Z, et al. (2008) Differential pathogenicity of SHIV infection in pig-tailed and rhesus macaques. Journal of medical primatology 37 Suppl 2: 13-23.

12. Bogers WM, Davis D, Baak I, Kan E, Hofman S, et al. (2008) Systemic neutralizing antibodies induced by long interval mucosally primed systemically boosted immunization correlate with protection from mucosal SHIV challenge. Virology 382: 217-225.

13. Demberg T, Florese RH, Heath MJ, Larsen K, Kalisz I, et al. (2007) A replication-competent adenovirus-human immunodeficiency virus (Ad-HIV) tat and Ad-HIV env priming/Tat and envelope protein boosting regimen elicits enhanced protective efficacy against simian/human immunodeficiency virus SHIV89.6P challenge in rhesus macaques. Journal of virology 81: 3414-3427.

14. Xu R, Srivastava IK, Greer CE, Zarkikh I, Kraft Z, et al. (2006) Characterization of immune responses elicited in macaques immunized sequentially with chimeric VEE/SIN alphavirus replicon particles expressing SIVGag and/or HIVEnv and with recombinant HIVgp140Env protein. AIDS research and human retroviruses 22: 1022-1030.
